# Accelerated Aging on the Compression Properties of a Green Polyurethane Foam: Experimental and Numerical Analysis

**DOI:** 10.3390/polym15071784

**Published:** 2023-04-03

**Authors:** Enio H. P. Da Silva, Silvio De Barros, André F. C. Vieira, Romeu R. C. Da Costa, Marcelo L. Ribeiro

**Affiliations:** 1 Aeronautical Engineering Department, São Carlos School of Engineering, University of São Paulo, São Carlos 13563-120, SP, Brazil; 2 CESI LINEACT, 44600 Saint-Nazaire, France; 3 Center for Mechanical and Aerospace Science and Technologies (C-MAST-UBI), Universidade da Beira Interior, R. Marquês D’Ávila e Bolama, 6201-001 Covilhã, Portugal; 4 Mechanical Engineering Department, Federal University of Technology—Paraná, Cornélio Procópio 86300-000, PR, Brazil

**Keywords:** bio-based polymer, polymer degradation, polyurethane foam, polymer aging

## Abstract

The aim of this work is to evaluate the changes in compression properties of a bio-based polyurethane foam after exposure to 90 °C for different periods of time, and to propose a method to extrapolate these results and use a numerical approach to predict the compression behaviour after degradation for untested conditions at different degradation times and temperatures. Bio-based polymers are an important sustainable alternative to oil-based materials. This is explained by the foaming process and the density along the material as it was possible to see in a digital image correlation analysis. After 60 days, stiffness was approximately decreased by half in both directions. The decrease in yield stress due to thermo-oxidative degradation had a minor effect in the foaming directions, changing from 352 kPa to 220 kPa after 60 days, and the transverse property was harshly impacted changing from 530 kPa to 265 kPa. The energy absorption efficiency was slightly affected by degradation. The simulation of the compression stress-strain curves were in accordance to the experimental data and made it possible to predict the changes in mechanical properties for intermediate periods of degradation time. The plateau stress for the unaged foam transverse to the foaming direction presented experimental and numerical values of 450 kPa and 470 kPa, respectively. In addition, the plateau stresses in specimens degraded for 40 days present very similar experimental and numerical results in the same direction, at 310 kPa and 300 kPa, respectively. Therefore, this paper presents important information regarding the life-span and degradation of a green PUF. It provides insights into how compression properties vary along degradation time as function of material operation temperature, according to the Arrhenius degradation equation.

## 1. Introduction

The price of the human desire to constantly improve its quality of life has been paid over the last centuries at the expense of our planet; if no actions are taken, the damages could be irreversible as the global population is now estimated to be 7.7 billion and it is expected to reach 9.5 billion by 2050 [1]. That means we must provide more food, crops and housing for all those people which would increase the damage to our land. However, by the means of science, we are now able to process more waste, to produce more food with less land and to develop bio-based and biodegradable materials, which is a promising path toward sustainability [2,3]. Plastics are responsible for around 10% of all generated waste in the world and they comprise 60–90% of marine litter. An alternative and more sustainable way to reduce the ecological damage that polymers cause is the development and application of bio-based materials. They have been the focus of many scientific studies because they cause a reduction in the use, production, and refinement of oil, consequently reducing greenhouse gas emissions [4,5].

Polyurethane foam (PUF) is an important plastic material used in many different industries such as automotive, furniture, aircraft, construction, etc. The lifespan of PUFs varies with the kind of exposure, and they can last from 3 to more than 50 years. For example, they present a lifetime of 15 years for refrigerator insulation. However, there is a lack of scientific publications regarding the lifespan of PUFs where their mechanical properties are relevant, such as sandwich panels [6]. Many studies regarding the synthesis, characterizations and applications of bio-based PUFs are being carried out every year due to its pairing characteristics with oil-based foams as well as its relative low cost and eco-friendly origin [7,8,9,10]. PUF is a two-phase material composed of a continuous polymer matrix and the gas in the discretely distributed cells. The cellular structure has a certain level of anisotropy due to the elongation of the cells towards the foaming direction during the expansion process. Therefore, the PUF mechanical properties in the expanding direction may present different values to those in the perpendicular direction [11]. The level of anisotropy varies with the density of the foam, where denser foams are often manufactured in high-pressure molds. This pressure tends to shape the cells into spheres, thus decreasing the anisotropy of the material [12,13,14]. There are many works regarding polyurethanes’ main properties, applications and reinforcements, but the amount of information regarding bio-based PUF properties is still incomparable to the one for oil-based PUF.

The current development, characterization and degradation of bio-based polyurethanes has been the goal of many researchers in recent years [8,15,16,17]. Lee et al. (2021) [18] developed bio-based PUFs by using a commercial castor oil (CO) as polyol as well as CO added with mercaptoethanol (COM) and α-thioglycerol (COT). The authors discovered that the added chemicals increased the viscosity of the mixture which increased the density of the foam by slowing and weakening the expansion. However, the compression strength of the COM and COT were substantially improved when compared to the CO foam, increasing from around 250 kPa to over 500 kPa and 550 kPa for the COM and COT, respectively. Maiuolo (2021) [12] synthesised high density bio-based PUFs from cellulose. They used a polyol-isocyanate ratio of 0.3/1 and added cellulose citrate (CC) as mechanical reinforcement in a CC-isocyanate ratio of 0.2/1. For foams with densities of 260 kg/m${}^{3}$ and 290 kg/m${}^{3}$ for the pure and reinforced foams, respectively, the compressive strength showed an enhancement from 2.03 MPa to 2.41 MPa, showing that the cellulose reinforcement was successfully applied in a cellulose-based bio-polyol. Moreover, their PUF presented anisotropy indexes (ratio between length and width of the cells) very close to 1, which represents an isotropic behavior characteristic to the high density foam they used.

Many authors have approached the numerical simulation of foams’ mechanical properties and the models can be easily divided according to the scale of the approach [19,20,21]. The main division is between direct (micro) models, which are based on the properties of the bulk material and the geometry of the cell sizes, and constitutive (macro) models based on a simpler element representing the homogenized foam properties [21]. However, none of them have approached the simulation of the temperature degradation on the compression properties of a bio-based foam, particularly in a non-isotropic way. He et al. (2019) [22] studied the degradation of tensile properties of a PUF for a brief period of 1 h at temperatures up to 200 °C. They applied an Abaqus UMAT subroutine to properly describe the elasto-plastic behavior of the foam under tensile loads. Their foam had a density of 150 kg/m³ and its elasticity modulus dropped from 22.8 MPa for the untreated foam to 15.7 MPa for the foam exposed to 200 °C.

The general compression behavior of foams can be divided in three main categories: elasticity of the cell walls; plastic deformation of the cell walls under constant load (plateau); densification hardening after the full collapse of the cells. In the linear elastic region, the stress and strain grow proportionally to each other and if the material is unloaded, it will restore its original configuration. This is commonly observed at strains ranging from 5% to 10%, moreover the slope of the stress–strain curve in the linear elastic region represents the foam’s modulus of elasticity. The long plateau region extends from the yield stress point, where the linear elastic region ends, up to the start of an exponential growth in stress. In this plateau region, the stress is almost constant as the strain increases, and it represents the failure of the cells being crushed. Moreover, since the size of the cells may be different throughout the foam, some cells will fail before others, which may cause a non-constant plateau (strain hardening). Furthermore, a stress hardening behavior may appear before fracture. It appears as an exponential stress growth after the cells are completely smashed and the foam’s density tends towards the polymer’s [23].

The plateau region is also the main factor responsible for the high energy absorption characteristic of the foams, since it is basically pure plastic deformation. The specific energy (work per unit of volume) is the area below the stress-strain curve, and it is calculated as follows:

(1.1) $$U=\int_{0}^{\varepsilon }\sigma \left(\varepsilon \right)d\varepsilon$$

furthermore, the efficiency of the energy absorption can be described as the ratio between the specific energy and stress. Efficiency of energy absorption is more suitable to represent the ability of a material to absorb energy, since it takes in consideration the amount of stress being applied. The energy absorption efficiency can be calculated using Equation (1.2):

(1.2) $$\eta =\frac{\int_{0}^{\varepsilon }\sigma \left(\varepsilon \right)d\varepsilon }{\sigma (\varepsilon )}$$

where *U* is the deformation energy, η is the energy absorption efficiency, and σ(ε) is the stress at its respective strain [23].

Marvi-Mashhadi et al. [24] carried out a high fidelity simulation of foam’s mechanical properties. The authors reported a divergence between numerical and experimental results due to the gap of two years between unloading/reloading tests and the monotonic ones as they have not considered the properties varying with the aging time. Lou et al. [25] studied the accelerated ageing of rubber foams regarding compression properties. However, they did not elaborate a numerical tool to simulate other results. Hence, there is a gap in the literature regarding the prediction of mechanical properties after ageing of cellular materials, especially towards bio-based and sustainable materials.

Therefore, the aim of this work is to report the temperature degradation of a bio-based polyurethane foam regarding their compression properties. Additionally, it will provide a numerical approach to simulate those properties for untested periods of degradation. Thus, the present work pushes the boundaries of the literature towards bio-based materials and their applications.

## 2. Materials

The raw materials of the PUF used in this work were bought from Kehl Company and are cataloged by the manufacturer as IC200 and KT1106-R for the isocyanate and polyol, respectively. The isocyanate is a Methylene Diphenyl Diisocyanate (MDI) with a density of 1.22 g/cm${}^{3}$ and viscosity between 170 and 250 mPa·s at room temperature. The polyol is a bio-based material from a blend of vegetable oils (mainly castor oil). Its density is 1.0 g/cm${}^{3}$ and its viscosity is around 3000 mPa·s.

### 2.1. Manufacturing Process

The density of the foam chosen for this work was 100 kg/m${}^{3}$ (0.1 g/cm${}^{3}$) because it was the smallest density in closed expansion that showed a good dimension standardization by filling every corner with the mold. Additionally, Linul et al. (2017) [26] reported that the best density regarding energy absorption is 100 kg/m${}^{3}$. A density around this value was also studied in the works of Liu et al. (2019) [27], Mazzuca et al. (2021) [28], and Iqbal et al. (2022) [29].

**Figure 1:**
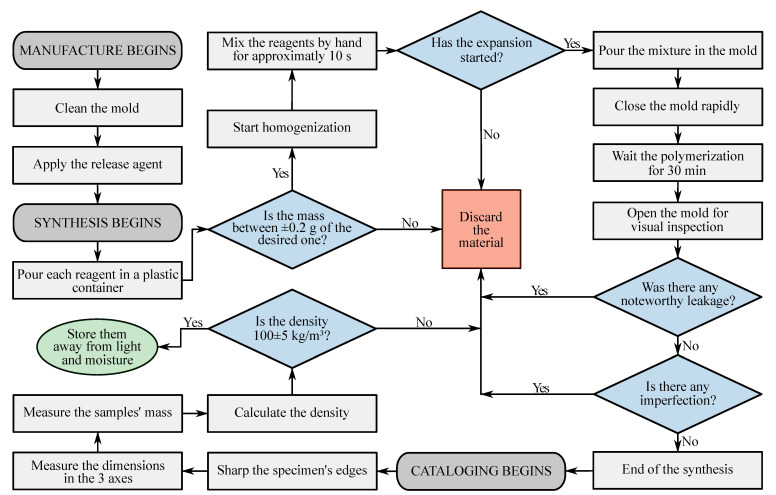
Flowchart of the PUF manufacturing process.

The raw materials were homogenized by hand with a mass proportion of 1:1 with 8 g of each material. In order to standardize the samples, a manufacturing optimal process was applied and it is described in the flowchart in Figure 1.

**Figure 2:**
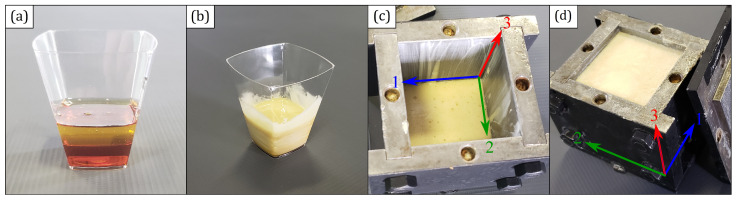
Synthesis process of the foam: (**a**) Polyol and isocyanate. (**b**) Hand-homogenized mixture. (**c**) Mixture poured into the mold where directions 3 and 1 represent the expanding and transverse ones, respectively. (**d**) Finished foam after expansion.

Specimens that did not fit the standardization criteria were discarded. A ground steel mold closed with screws was used to contain the expansion. Figure 2 shows the manufacturing process of the foam from both components to the finished cube. The expanding direction was marked and the specimens were kept away from light, moisture and heat until the necessary amount of samples were manufactured, then, they were all together exposed to accelerated degradation.

### 2.2. Ageing Process

**Figure 3:**
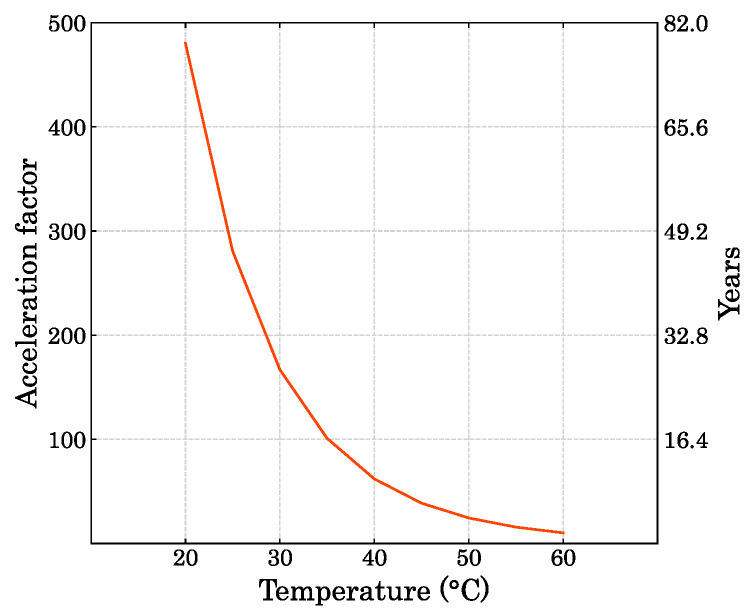
Acceleration factor and ageing estimation per operation temperature.

The Arrhenius equation describes the rate of a chemical reaction based on the temperature and is written as shown in Equation (2.1), [30,31]:

(2.1) $$K=A\exp\left(\frac{-E_{a}}{RT}\right)$$

where *K* is the Arrhenius degradation rate constant of the desired phenomenon; *A* is a pre-exponential and experimental factor; $E_{a}$ is the activation energy for the respective phenomenon; *R* the universal gas constant (8.3145 J/K/mol); and *T* is the temperature in Kelvin [K]. Furthermore, the $E_{a}$ can be obtained from a modified Arrhenius equation that relates two different degradation rates with their respective temperatures as shown in Equation (2.2):

(2.2) $$\ln\frac{K_{2}}{K_{1}}=\frac{E_{a}}{R}\left(\frac{1}{T}-\frac{1}{T^{\prime }}\right)$$

where $T^{\prime }$ is the temperature in the accelerated environment (elevated temperature) and *T* is the operation temperature of the material. The $E_{a}$ represents the minimum amount of energy a sample must posses in order to undergo a transformation. Hence, by increasing the thermal degradation activation energy, the material thermal stability also increases. [32,33]. Consequently, using the obtained activation energy it is possible to estimate the acceleration factor by modifying the Arrhenius equation as shown in Equation (2.3) [34]:

(2.3) $$AF=\exp\left[\frac{E_{a}}{R}\left(\frac{1}{T}-\frac{1}{T^{\prime }}\right)\right]$$

where AF is the acceleration factor that represents the time ratio of the reaction under different temperatures. In the work conducted by Berardi (2019) [35], a polyurethane foam was exposed for 4.5 months at 70 °C. The operation temperature and activation energy was assumed to be 20 °C and 60 kJ/mol, respectively. The author’s acceleration factor was around 29 which allowed the simulation of a degradation at room temperature over more than a decade [36]. Moreover, Sandia National Laboratories [37] elaborated a report where an Arrhenius activation energy was around 78 kJ/mol. Additionally, Lee et al. (2018) [38] reported an activation energy value of 76 kJ/mol for a compression spring constant. Thus, in order to simulate degradation for long periods of time at room temperature, in the present work a bio-based PUF has been kept at 90 °C for 10, 20, 30, 40, 50, and 60 days, which considering an average activation energy of 70 kJ/mol will represent an acceleration factor as a function of the operation temperature as shown in Figure 3. Here, it is shown how the AF changes according to the desired operation temperature (smaller than the accelerated degradation temperature of 90 °C), since if it gets closer to 90 °C, the acceleration is going to be smaller up to 1 if the temperatures are the same (that means no acceleration).

Thus, the degradation in this work can simulate the degradation of the PUF for several decades depending on the operation temperature. For instance, if the temperature the material will be exposed to in real life is 40 °C, the acceleration factor will be approximately 62. Therefore, as the time of exposure at elevated temperature in this work was 60 days, the degradation of compression properties of those 60 days at 90 °C will represent a degradation of around 10 years (60·62/365) at 40 °C.

## 3. Methods

The ageing process was carried out in a computer-controlled convection oven by Cienlab that kept the temperature constant for the whole time. After degradation, specimens presented a little dimensional changing, losing the adequate parallelism. Thus, they were sanded back to their original shape. The mass change (Equation (3.1)) in the aging process considered the mass before the sanding whereas the mass loss was only due to degradation:

(3.1) $$ml\%=100\cdot \frac{m_{0}-m_{d}}{m_{0}}$$

where ml% is the mass loss percentage, $m_{0}$ is initial mass, $m_{d}$ is the mass after the degradation process [31,39].

### 3.1. Compression Test

The D1621-16 standard of the American Society for Testing and Materials (ASTM) [40] specifies the test method for compressive properties of rigid cellular plastics. Therefore, it is suitable for the application in this work and widely applied for polymeric foam characterizations. It has been used in the works of Liu (2019) [27], Liu (2020) [41], and Li (2021) [42]. They performed compression tests in polyurethane foam, poly(vinyl chloride) foam and polystyrene foam, respectively.

According to the standard, the specimens should have a cross section area of at least 25.8 cm${}^{2}$, and the height should not exceed the width and depth. Therefore, considering the cross section as a square, the width and depth of the specimens should be at least 50.8 mm. In that way, the dimensions adopted in this work were 50.8 × 50.8 × 50.8 mm${}^{3}$.

Considering the foam to be a transversely isotropic cellular material as the results presented by Tita (2012) [43] and Tagarielli (2005) [44], the compression tests were carried out in two directions for each ageing time, the first one being the expansion direction (upwards) and the second being orthogonal to the expansion. Five test specimens were created for each direction for a total of ten test specimens for each ageing time.

**Figure 4:**
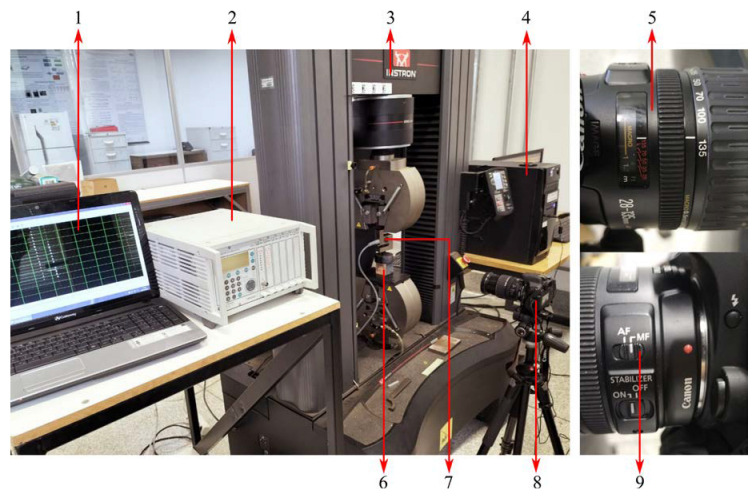
Setup applied for the compression tests labeled as follows: 1. MGCplus Assistant software to show the strength data acquired from the HBM. 2. HBM MGCplus AB22A data acquisition system connected to the load cell. 3. Instron UTM 5985 controlling the test speed. 4. Computer controlling the UTM by the Bluehill software and the camera by the software EOS Utility Version 3 (2015). 5. Canon Macro lens model EF 28–135 mm IS with minimal focal distance of 0.5 m. 6. Test specimen. 7. 20 kN Load cell from Vicere model ST2K. 8. Canon EOS Rebel T6i placed 0.55 m from the sample on a tripod stand. 9. The focus was set to manual to optimize the quality.

The tests were carried out in a universal testing machine with test speed of 5 mm/min, which represents 10% of the specimen initial height per minute. The strain values were obtained with the Digital Image Correlation (DIC) technique using the software GOM Correlate. In order to do so, one side of each specimen was painted with a black background with a pattern of white dots over it, so the software can recognize the deformation by comparing the position of the white dots. The pictures were taken every 5 s with a resolution of 6000x4000 pixels. Room lighting was good enough to provide a good pattern recognition on GOM Correlate. The test setup is shown in Figure 4. Four transverse (two for each plane orthogonal to the load-normal plane) extensometers, 45 mm long, were placed on the image of the specimens for the acquisition of the true transverse strain. The longitudinal strain was calculated from the displacement (acquired from the DIC) of the machine’s cross head.

The strength data were obtained every 0.2s and a 12 degree polynomial fitting using Python Scipy Optmize Curve_fit was used to extract 100 values of strength and time that perfectly fit the raw data. Then, the same procedure was performed with the Digital Image Correlation (DIC) strain data (raw values every 5 s) to obtain 100 data sources of strain/displacement and time. Finally, strength and strain/displacement data were matched as they represent the same test time.

### 3.2. Numerical Implementation

The prediction of the degradation of the mechanical behavior was implemented using an Abaqus UMAT user subroutine. The constitutive model used in this work was based on the transversely isotropic model proposed by Tagarielli [44] that was derived from the generalized Hill’s effective stress [45]:

(3.2) $$C^{-1}=\left[\begin{array}{cccccc} \frac{1}{E_{1}} & -\frac{\nu_{12}}{E_{1}} & -\frac{\nu_{13}}{E_{3}} & 0 & 0 & 0\\ -\frac{\nu_{12}}{E_{1}} & \frac{1}{E_{1}} & -\frac{\nu_{13}}{E_{3}} & 0 & 0 & 0\\ -\frac{\nu_{13}}{E_{3}} & -\frac{\nu_{13}}{E_{3}} & \frac{1}{E_{3}} & 0 & 0 & 0\\ 0 & 0 & 0 & \frac{2(1+\nu_{12})}{E_{1}} & 0 & 0\\ 0 & 0 & 0 & 0 & \frac{2(1+0.5\nu_{13}+0.5\nu_{31})}{E_{3}} & 0\\ 0 & 0 & 0 & 0 & 0 & \frac{2(1+0.5\nu_{13}+0.5\nu_{31})}{E_{3}} \end{array}\right]$$

where $E_{1}$ is the stiffness in the transversely isotropic directions and $E_{3}$ is the reference stiffness value; $\nu_{12}$ and $\nu_{13}$ are the two Poisson’s ratio for uniaxial loading. The shear properties for the transversely isotropic model were applied as calculated by Xu (2022) [11]. The stress was calculated using the Jaumann objective stress rate in Equation (3.3) [46]:

(3.3) $$\overset{\nabla }{\sigma }=2GD^{e}+\lambda \mathrm{Tr}\left(D^{e}\right)I$$

where $\overset{\nabla }{\sigma }$ is the Jaumann stress rate; *G* is the shear modulus of the foam; $D^{e}$ is the elastic strain rate matrix; λ is the Lamé’s first parameter; and *I* is an identity matrix. The elastic strain rate matrix is equal to the total strain rate matrix ($D^{e}=D$) if the yield criterion has not been satisfied. Otherwise, the strain rate decomposition followed the rule ($D=D^{e}+D^{p}$). Then, the material stress rate can be calculated by Equation (3.4):

(3.4) $$\dot{\sigma }=\overset{\nabla }{\sigma }+W\sigma +\sigma W$$

where $\dot{\sigma }$ is the stress rate and *W* is the spin matrix. Both spin and total strain rate matrix were calculated by the deformation gradient given by Abaqus as shown in the following equations:

(3.5) $$L=\dot{F}F^{-1}$$

(3.6) $$D=\frac{1}{2}\left(L+L^{T}\right)$$

(3.7) $$W=\frac{1}{2}\left(L-L^{T}\right)$$

where *F* is the deformation gradient; $\dot{F}$ is the deformation gradient rate; and *L* is the velocity gradient. Furthermore, in order to calculate the effective stress for the yield criterion, a transversely isotropic adaptation from the von Mises criterion was adopted as shown in Equation (3.8):

(3.8) $$\bar{\sigma }=\sqrt{\sigma^{T}Q\sigma }$$

where *Q* is the yield transversely isotropy matrix that represents the ratio of yield strength relative to a reference direction (dir. 3 in this case) as shown in Equation (3.9):

(3.9) $$Q=\left[\begin{array}{cccccc} B^{2} & -\frac{C^{2}}{2} & -\frac{D^{2}}{2} & 0 & 0 & 0\\ -\frac{C^{2}}{2} & B^{2} & -\frac{D^{2}}{2} & 0 & 0 & 0\\ -\frac{D^{2}}{2} & -\frac{D^{2}}{2} & 1 & 0 & 0 & 0\\ 0 & 0 & 0 & E^{2} & 0 & 0\\ 0 & 0 & 0 & 0 & F^{2} & 0\\ 0 & 0 & 0 & 0 & 0 & F^{2} \end{array}\right]$$

the five constants are defined by: $B^{2}=Y_{33}^{2}/Y_{11}^{2}$, $C^{2}=2B^{2}\nu_{12}^{p}$, $D^{2}=2\nu_{31}^{p}=2\nu_{32}^{p}$, $E^{2}=Y_{33}^{2}/Y_{12}^{2}$, $F^{2}=Y_{33}^{2}/Y_{23}^{2}=Y_{33}^{2}/Y_{31}^{2}$, where $Y_{11}$ and $Y_{33}$ are the uniaxial loading flow stresses in directions 1 and 3, respectively. $Y_{12}$ and $Y_{31}$ are the shear flow stresses in their respective planes, and $\nu_{ij}^{p}$ is the plastic Poisson’s ratio. For this model, the yield function is represented by Equation (3.10):

(3.10) $$f=\sqrt{\sigma^{T}Q\sigma }-Y_{33}$$

if after the elastic trial the yield function returns f>0, the plasticity calculation begins in order to bring *f* back to 0. Moreover, if the plastic strain rate is assumed to be normal to the yield surface (associated flow), the model results in a plastic strain rate as described in Equation (3.11) where the df/dσ represents the mapping of the effective strain rate. Furthermore, the plastic strain rate matrix ($D^{p}$) can be obtained as shown in Equation (3.12):

(3.11) $${\dot{\varepsilon }}^{p}={\dot{\bar{\varepsilon }}}^{p}\frac{df}{d\sigma }={\dot{\bar{\varepsilon }}}^{p}\frac{Q\sigma }{\bar{\sigma }}$$

(3.12) $$D^{p}=\left[\begin{array}{ccc} {\dot{\varepsilon }}_{11}^{p} & 0 & 0\\ 0 & {\dot{\varepsilon }}_{22}^{p} & 0\\ 0 & 0 & {\dot{\varepsilon }}_{33}^{p} \end{array}\right]$$

applying the strain decomposition it is possible to calculate the Jaumann stress rate (Equation (3.3)) and then the stress rate for plasticity (Equation (3.4)). The interaction between Abaqus and the calculation performed by the UMAT is represented in Figure 5.

The degradation of the compression properties was implemented considering the stress to be a function of time exposure in the convection oven. Thus, as shown in Equation (2.3) it will represent a degradation over longer periods of time for any application temperature under 90 °C. Therefore, the stiffness matrix *C* shown in Equation (3.2) obtains the shape of C(t), as well as the hardening function for the plasticity implementation $H({\bar{\varepsilon }}^{p},t)$.

The element used in this simulation was a C3D20 20-node quadratic brick with 1000 elements. Two parallel rigid plates were positioned at the top and bottom bounders of the cube with a friction coefficient of 0.001, one plate was clamped and the other was displaced 35 mm in the desired direction to simulate the same procedure carried out in the experimental tests. This load mechanism is quite similar to the one used by Linul (2017) [26]. This macro approach represents with certain precision what happens throughout the whole material. Micro behavior approaches are more indicated when aiming to represent the stress gradient along the material, similarly to Voronoi diagram seeded cells and Laguerre tessellation, as shown in the works of Hössinger-Kalteis (2022) [47] and Shiravand (2022) [20], respectively. However, the micro failure behavior of the foam is not the emphasis of this work, instead, it aims to predict the degradation of a bio-based material over time and for this reason the macro approach was considered more reasonable.

## 4. Results

**Figure 6:**
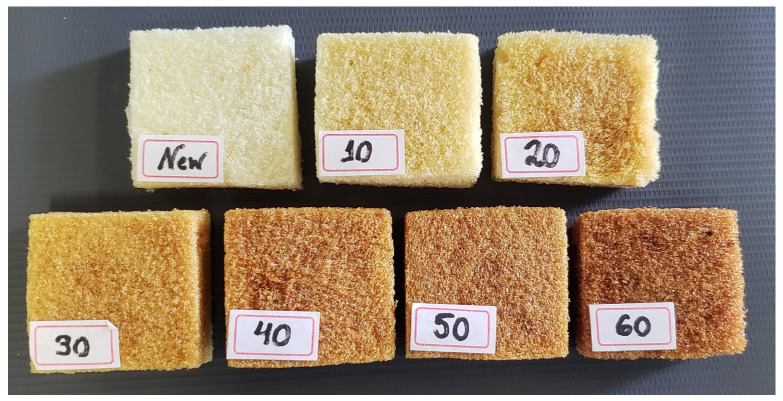
Difference in the PUF color after the ageing process.

After the ageing process, the color of the foam became darker as the ageing time increased. Figure 6 shows the inner part of the specimens after each accelerated degradation time.

**Figure 7:**
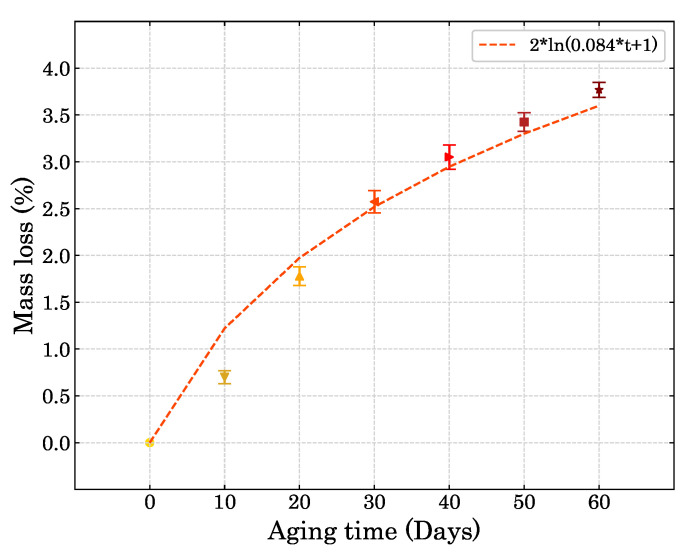
Mass loss of the compression specimens after the ageing process.

The mass loss for ageing times up to 60 days was studied. Figure 7 shows the progression in mass change. A logarithmic mass loss fitting was adopted in the shape of ml=2ln(0.084t+1), as *t* is the number of days in the convection chamber.

### 4.1. Compression Test Results

The compression tests were carried out in two directions, where the direction of expansion was set as the main one and named Direction 3 (Dir3). The directions transverse to the expansion were called Direction 1 (Dir1) and Direction 2 (Dir2). Moreover, Dir1 and Dir2 were considered to be equal, since there is no reason for them to be different based on the manufacturing process and many cellular material models in the literature have made the same consideration [44,48,49]. The convention adopted in this work follows the classical transverse isotropic formulation, where $E_{1}=E_{2}\neq E_{3}$.

**Figure 8:**
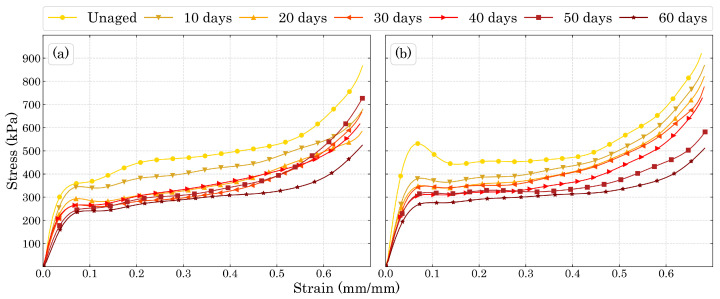
Compression stress-strain curves for (**a**) Dir3 and (**b**) Dir1.

Figure 8 shows the compression stress-strain results for the degraded foams in both directions studied in this work. The decrement in stiffness and compression strength were well pronounced as the degradation time increased.

**Figure 9:**
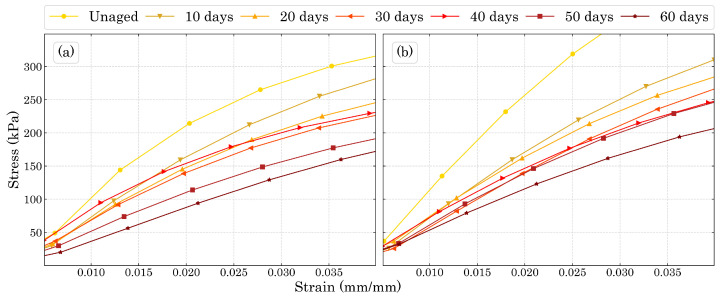
Initial compression stress-strain curves for (**a**) Dir3 and (**b**) Dir1.

The three compression regions are explicit in Figure 8, where the elastic region reached approximately the same strain for all the ageing times in each direction. While for Dir3 the elastic region reached a strain of around 6%, for Dir1 it extended to about 7%. The plateau region for both directions ended at around 50% of strain. Figure 9 shows the beginning of the stress-strain curve in order to better represent the elastic region of the materials.

**Figure 10:**
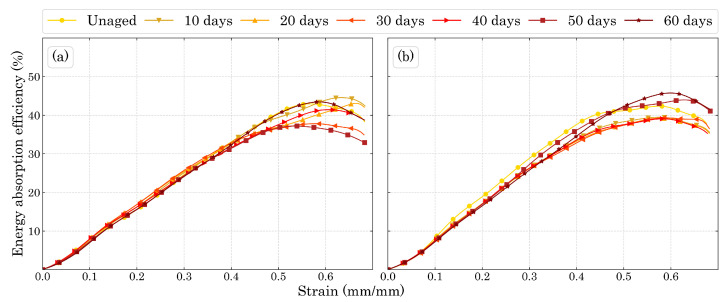
Energy absorption efficiency for (**a**) Dir3 and (**b**) Dir1.

The decreasing stiffness with the increasing ageing time as well as the mass loss presented by the specimens show that the damage caused by temperature is most likely to be due to sisions of the polyurethane molecule than to the formation of cross-links between molecular chains that would increase the stiffness of the material. In the work of Mohammadi et al. [50] it was shown that during ageing at high temperatures, there is both molecular sision along the macromolecules and further cross-links between the molecular chain which is responsible for PUF’s embrittlement. Regarding the plastic region, the relationship between the plateau and the foam’s energy absorption efficiency is shown in Figure 10 as the densification (end of plateau) decreases the efficiency of energy absorption, therefore, this bio-based PUF as plastic energy absorption material works better for strains up to 50∼60%.

The higher compression and plateau stress of the unaged foam for both directions could represent a lower energy absorption efficiency as it is inversely proportional to the stress as shown in Equation (1.2). However, there were no noteworthy differences among the samples for each ageing time. Thus, it means that the ageing process did not harm nor increase the energy absorption efficiency, where the higher strength of the unaged foam also showed higher toughness.

**Figure 11:**
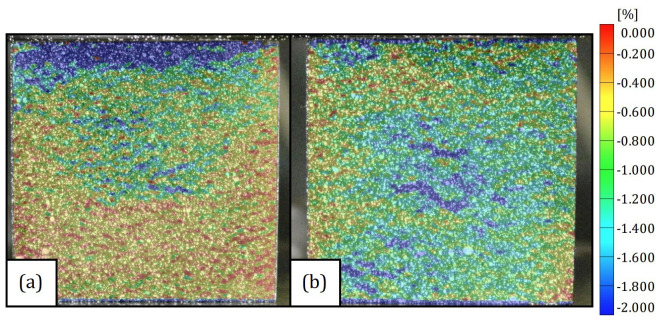
DIC strain field for (**a**) Dir3 and (**b**) Dir1.

The difference in the mechanical properties between both directions can be easily explained by their DIC strain fields shown in Figure 11. The strain field in Dir3 shows a concentration of strain in the top of the specimens, which represents the last region of the mold filled with polymeric material. Furthermore, this top region is expected to have a smaller density than the rest of the specimen. Thus, it matches the strain field shown in Figure 11a. Since the material is being treated as transversely isotropic, Dir1 should exhibit a strain field more homogenized which is exactly as can be seen in Figure 11b.

The hardening plateau presented in Figure 8a can also be explained by the DIC images, since the size of the cells is expected to be different along Dir3, the cells on the top of the specimens will fail before the ones below them. Thus, while the lower cells have not failed, the stress necessary to change the configuration of the sample slightly increases with the strain, which is in accordance to the behaviour of a typical cellular material strain hardening [23].

### 4.2. Numerical Prediction Results

By applying the Tagarielli yield criterion as function of the time of exposure in the convection chamber, it was possible to estimate the compression behavior of the bio-based PUF under untested ageing periods of time. The simulated stress-strain curves can properly fit the experimental ones depending on the amount of fitting parameters used in the σ(t) and E(t). In order to simplify and reduce the number of parameters and present a typical Arrhenius degradation behavior of a negative radical function of time [34], two functions were adopted in the form of Equations (4.1) and (4.2):

(4.1) $$\bar{\sigma }=\left(s_{1}t^{s_{2}}+s_{3}\right)$$

(4.2) $$E=(e_{1}t^{e_{2}}+e_{3})$$

additionally, the plasticity tangent modulus (*H*) was obtained by the derivative of the two variable functions represented by Equation (4.3), which was the optimal shape for the $\bar{\sigma }\left({\bar{\varepsilon }}^{p},t\right)$ function based on the fitting of the experimental data.

(4.3) $$\bar{\sigma }=\frac{{\left(h_{1}t+h_{2}\right)}^{h_{3}}}{{\left(h_{4}{\bar{\varepsilon }}^{p^{2}}+h_{5}t+h_{6}\right)}^{h_{7}}}$$

**Figure 12:**
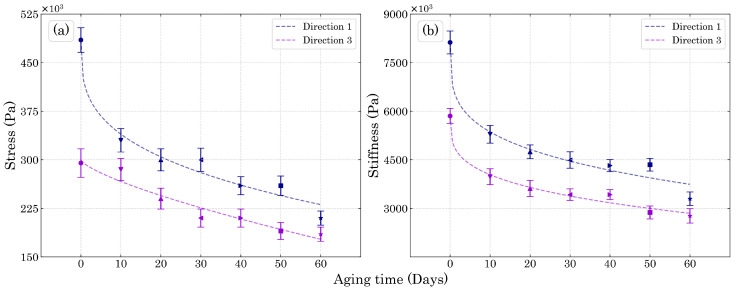
UMAT properties of (**a**) yield stress and (**b**) stiffness for Dir1 and Dir3 with their respective fitting curves.

It is important to realize that for large deformation theory, Abaqus uses nonlinear geometry configuration Thus, the strain values calculated by the software are always logarithmic strains. Hence, the strain values used in the stress-strain curves for the fitting parameters must be logarithmic, unlike the most common way to present compression values (engineering strain) since compression logarithmic strains can go beyond 100%. The fitting tool was the Python Scipy Optimize Curve_fit using the non-linear least square method. The fitting curves for the yield stress and compression elasticity modulus are shown in Figure 12, where the yield stress was obtained at the end of linearity. Furthermore, the fitting parameters obtained in this work are shown in Table A1 of Appendix A.

**Figure 13:**
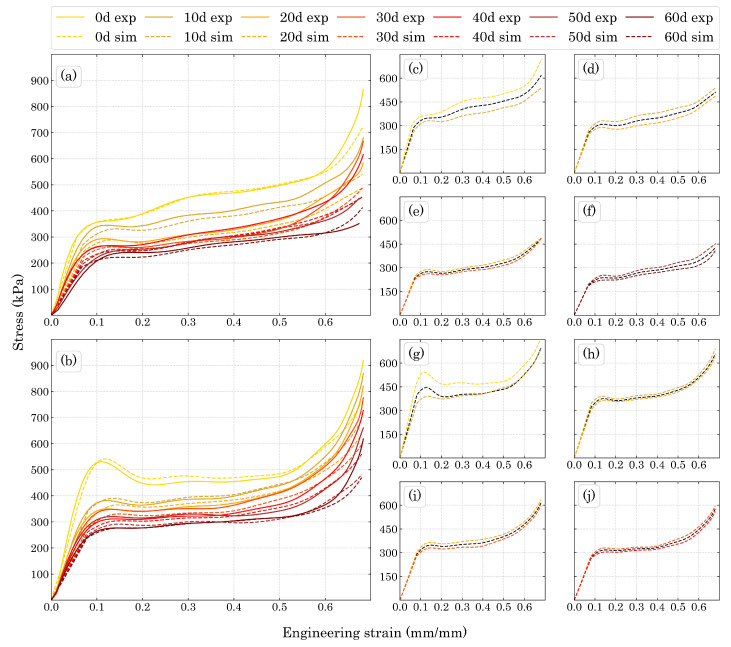
Numerical and experimental results with their respective degradation time for (**a**) Dir3 and (**b**) Dir1. Simulation for Dir3 with ageing period of (**c**) 5, (**d**) 15, (**e**) 25, and (**f**) 55 days and simulation for Dir1 for aging periods of (**g**) 5, (**h**) 15, (**i**) 25, and (**j**) 35 days.

Figure 12a,b show that the fitting functions were adequate for the experimental compression test results. Thus, with their application in the subroutine it was possible to predict the compression properties over intermediary untested periods of time for both Dir3 and Dir1. As shown in Figure 13, which compares the experimental results with the numerical ones in Figure 13a,b for Dir3 and Dir1, respectively. The difference between the experimental and numerical values are very close given to the stiffness fitting function, as the Dir3 fitted compression elastic modulus represented by the curve in Figure 12b presents higher values for the 50 and 60 days aged foam than the experimental values for those ageing periods. Thus, it was expected that the linear elastic inclination for those numerical curves would be higher than their respective experimental curves and it is exactly what is shown in Figure 13a. However, those differences were quite small and considering the simplicity of the fitting functions the numerical results were rather accurate. The same explanation can be attributed to the yield difference for the Dir3 10 days aged foam. The plasticity *H* modulus were able to describe most of the plasticity behavior of the foams, especially up to the maximum energy absorption region.

Furthermore, Figure 13c–f present the Dir3 simulation for untested ageing periods showing that the parameters work well for any time between 0 and 60 days. Moreover, since the tangent modulus difference is smooth between the experimental curves for Dir3, the 5 days curves practically represents a mean curve between the 0 and 10 days aged foam and the same behavior can be observed for the following ones in Dir3. Figure 13g–j also show simulations for untested aging times but for Dir1. However, as seen in Figure 13g, which represents 5 days of accelerated aging, the curve does not represent an average of 0 and 10 days, instead, it grows quite as expected until the yield point, but then, in plasticity, the stress reaches and basically follows the 10 days curve. This can be explained by the difference in the tangent modulus behavior between the unaged and the 10 days aged foam. For the unaged foam there is a noteworthy decrement in stress before the beginning of the plateau while for the 10 days aged foam the decrement was almost negligible, thus, the 5 days aged simulation inherited an average behavior between the 0 and 10 days tangent modulus, presenting a significant, yet smaller than the unaged, decrement in stress before the plateau, which was enough to place it just above the 10 days aged foam stress-strain curve. This difference in *H* was high enough to influence just the 5 days simulation. For the others, the behavior was basically an average stress, which means that the parameters for Dir1 were all adequate and represented a plausible simulation of the accelerated ageing process.

The maximum stress before the beginning of the plateau region ($\sigma_{max}$) can be considered almost the same as the plateau stress ($\sigma_{pl}$) for all the Dir1 results except the unaged foam, where there is a significant stress drop before the plateau begins and it was well described by the unaged simulation. As expected, Dir3 numerical results showed a hardening plateau, as discussed in Section 4.1, matching the experimental behavior. Such characteristic can be simulated for a large range of ageing times as shown by Figure 13c–f.

Table 1 summarizes the numerical and experimental plateau results. The numerical $\sigma_{pl}$ for all the Dir1 ageing times were very close to the respective experimental results, except for the 50 days aged foam where plateau hardening inclination ($H_{pl}$) and maximum energy absorption strain occurred ($\varepsilon_{\eta_{max}}$), since the $\sigma_{pl}$ is not constant. The $H_{pl}$ represents the hardening plateau more characteristic of the Dir3 behavior and shows that the model properly describes this phenomenon since small differences in the numbers do not interfere at the $\varepsilon_{\eta_{max}}$ value.

This numerical simulation is important due to the Arrhenius acceleration factor shown in Figure 3 which allows the simulation of the degradation under practical applications. Thus, the years of operation exhibited in Figure 3 changes depending on the time of exposure in the accelerated ageing environment and the simulation of a specific ageing time can represent with good accuracy the degradation in the compression properties of the bio-based foam for the desired operation time and temperature.

## 5. Conclusions

This paper investigated the behavior of a bio-based PUF when exposed to a temperature of 90 °C for periods of 10, 20, 30, 40, 50 and 60 days, focusing on the compression properties. Additionally, this work aims to provide a numerical tool to predict the changes in those properties for untested periods of time. In order to do so, compression tests were carried out and numerical simulations were made using an Abaqus UMAT subroutine. The exposure of the foam to elevated temperature caused a variation in the foam’s color, geometry and mass. In addition, after comparing the mechanical compression behavior of the unaged foam with the ones exposed to 90 °C, a decrement was detected in stiffness and yield stress. Furthermore, this decrement was noticed after each interval of time in a behavior much like a negative radical function. This function was fitted in the degradation curve and provided degradation parameters to be used in the simulations. The implementation of these properties in UMAT proved to be promising, as the model was able to represent the experimental results with a good precision. That means that the fitting functions applied in this work are important information that can represent how the compression properties change over time. In addition, an estimation for the mechanical properties of the PUF in different temperatures can be made by calculating the acceleration factor for a desired working temperature by the Arrhenius equation. Therefore, the goals of this work have been achieved as it provides experimental data and a numerical tool to simulate the thermo-oxidative degradation of the foam, which is a key piece of information for the growing range of application of bio-based materials.

## Figures and Tables

**Figure 5:**
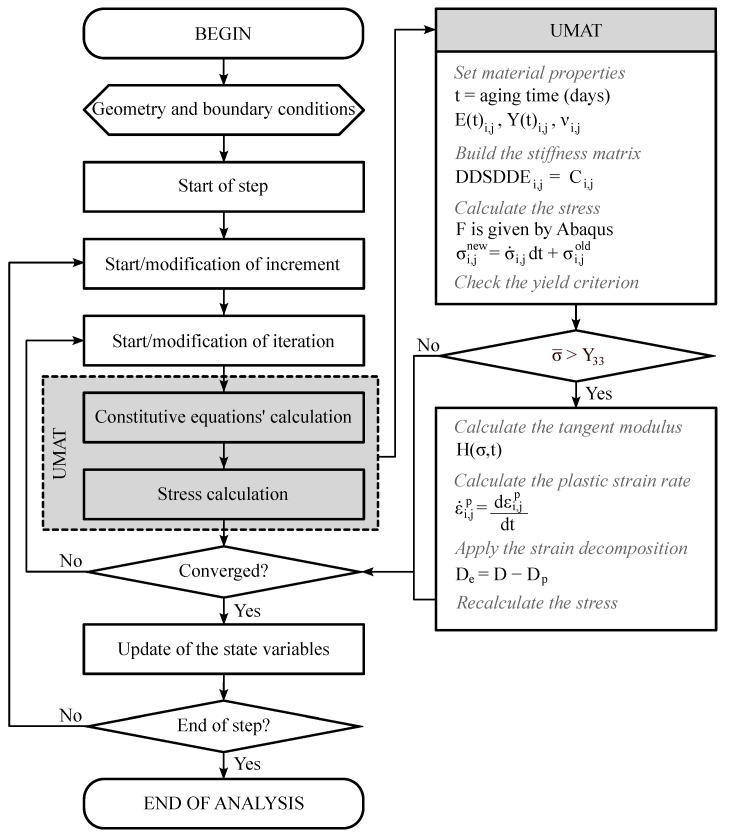
Flowchart of the UMAT application on Abaqus Standard.

**Table 1:** Comparison between numerical and experimental results for each tested ageing period.

Ageing	Dir3				Dir1			
	$H_{\mathrm{pl}}$ (kPa)		$\varepsilon_{\eta_{\max}}$ (mm/mm)		$\sigma_{\mathrm{pl}}$ (kPa)		$\varepsilon_{\eta_{\max}}$ (mm/mm)	
	Exp ^1^	Num ^1^	Exp	Num	Exp	Num	Exp	Num
Unaged	367	367	0.56	0.61	460 ^2^	480 ^2^	0.57	0.55
10 days	300	283	0.64	0.67	380	390	0.56	0.58
20 days	267	220	0.67	0.67	355	370	0.57	0.61
30 days	235	185	0.56	0.58	355	335	0.58	0.58
40 days	330	280	0.61	0.66	320	310	0.58	0.57
50 days	225	285	0.54	0.60	330	295	0.60	0.57
60 days	180	215	0.58	0.62	290	290	0.56	0.61

^1^ The inclination $H_{pl}$ was calculated for strain values between 20% and 50%. ^2^ The $\sigma_{pl}$ was significantly different from the $\sigma_{max}$ before the plateau.

## Data Availability

The detailed data presented in this work can be provided under reasonable request.

## Abbreviations

The following abbreviations are used in this manuscript:

PUF	Polyurethane foam
DIC	Digital image correlation
Dir3	Foaming (expansion) direction
Dir1	Direction transverse to the expansion

## Appendix A

Fitting parameters from the Python scipy.optmize.curve_fit function used in the Abaqus UMAT subroutine for the yield strength, elastic and tangent modulus.

**Table A1:** Fitting parameters used in the UMAT subroutine.

Parameter	Direction 1	Direction 3
$s_{1}$	−7.030 × $10^{4}$	−6.143 × $10^{3}$
$s_{2}$	3.132 × $10^{-1}$	7.307 × $10^{-1}$
$s_{3}$	4.842 × $10^{5}$	2.996 × $10^{5}$
$e_{1}$	−1.523 × $10^{6}$	−9.450 × $10^{5}$
$e_{2}$	2.579 × $10^{-1}$	2.823 × $10^{-1}$
$e_{3}$	8.119 × $10^{6}$	5.849 × $10^{6}$
$h_{1}$	−8.608 × $10^{-4}$	−5.786
$h_{2}$	1.551	1.006
$h_{3}$	1.277 × $10^{1}$	7.202 × $10^{2}$
$h_{4}$	−2.756 × $10^{-1}$	−6.687 × $10^{-2}$
$h_{5}$	−9.166 × $10^{-5}$	−3.947 × $10^{-3}$
$h_{6}$	4.879 × $10^{-1}$	7.560 × $10^{-1}$
$h_{7}$	5.895 × $10^{-1}$	5.375
